# Perceptions and Liking Distortion from Information about the Nutritional Upgrades in Biofortified Seafood Products

**DOI:** 10.3390/foods11182808

**Published:** 2022-09-12

**Authors:** Greta Castellini, Fosca Vezzulli, Milena Lambri, Gabriele Sacchettini, Guendalina Graffigna, António Marques, Ettore Capri

**Affiliations:** 1 EngageMinds Hub, Consumer, Food & Health Engagement Research Center, Università Cattolica del Sacro Cuore, 29122 Piacenza, Italy; 2 Department for Sustainable Food Process (DiSTAS), Università Cattolica del Sacro Cuore, 29122 Piacenza, Italy; 3 Aeiforia s.r.l., Loc. Faggiola 16, 29027 Gariga di Podenzano, Italy; 4 Division of Aquaculture and Upgrading (DivAV), Portuguese Institute for the Sea and Atmosphere (IPMA), 1749-077 Lisbon, Portugal

**Keywords:** consumer engagement, fish farming, food fortification, willingness to pay, sensory

## Abstract

(1) Background: As biofortified fish meat is becoming increasingly available, the use of supplements within fish feed may impact consumers’ perceptions and their willingness to pay (WTP) for the product. This study focused on evaluating the sensory liking of, and WTP for, fish produced with fortified feed while understanding the role played by the acquired information on fish fortification. (2) Methods: Hedonic ratings and WTP were measured in an experimental bid. The participants (n = 91) were asked to rate pleasantness and WTP during two different rounds: (i) appearance–information–tasting and (ii) appearance–tasting–information. A total of three fish species (carp, seabream, and trout) were presented to the consumers as being either fortified (with iodine, selenium, and omega-3 fatty acids) or conventional products. (3) Results: For pleasantness, no significant differences were found between the fortified and conventional fish. In contrast, substantial differences emerged when information regarding the products was provided. Providing the relevant information before tasting affected how much the consumers liked the conventional fish, resulting in a preference for it over the fortified fish. Additionally, consumers are willing to pay more for fortified fish, especially when information with respect to fortification is available. Nevertheless, when information about fortification was provided before tasting, the consumer’s expectations were not fulfilled. (4) Conclusions: The outcomes of this study clearly indicate that the presence of relevant information impacts how much people like fortified versus conventional fish, as well as their WTP.

## 1. Introduction

An innovative solution to increase the nutritional value of, and the toxic metal accumulation in, fish meat is to switch from a traditional supply to sustainably farmed products [1]. Indeed, farmed species can achieve a high standard of quality compared to wild specimens due to limited environmental spoilage and the use of feeds with bioactive supplements, which are able to increase the health benefits of the final product [2]. Such innovative management practices improve food quality, including its safety, as well allowing for sustainability goals, such as the Sustainable Development Goals 3, 12, 14, and 17, to be more easily achieved [https://sdgs.un.org, accessed on 31 August 2020]. Several studies on farming systems and types of habitats paired with studies on different diets and supplements given to targeted farm fish species [3,4,5,6] have already proven the efficiency of aquaculture to reduce stress and stress-related loss of quality in meat, improve nutrient utilization, and modulate diet to cope with the metabolic needs of fish species [2]. Fish farming also reduces the risk of toxic metals and organochlorine pesticides from being ingested by fish and accumulating in their meat, thus limiting consumer exposure to these contaminants through fish consumption [7].

As with many classes of foodstuffs, such as cereals, grains, milk, dairy, and plant-based products, fish meat is also becoming increasingly available in biofortified versions [8]. In this regard, several studies have been conducted, evaluating different feeds and supplements for fish nutrition using natural and sustainable ingredients to improve fish production, increase its nutritional value, and strengthen the industry and consumer acceptance. Peculiar examples of fortification include the use of brewery subproducts, seaweeds, microalgae, vegetables, and flowers in fish feeds [9,10,11,12,13,14,15,16]. Some studies focused on fortified fish consuming feeds that were rich in digestible proteins, vitamins (A, D3), trace minerals (iodine, selenium) and n-3 long-chain polyunsaturated fatty acids as a source of high-quality lipids and seaweed extracts as a natural antioxidant [17,18,19].

Fish farming and biofortification represent an efficient way to improve fish meat quality. Special attention must be paid when considering the diets of vulnerable segments of the population, such as the elderly and infants [20,21]. Moreover, consumers have demonstrated their growing interest in fortified and biofortified foods [22] thanks to the perceptions of increased wellness from these products [23,24]. 

However, the use of supplements in fish feed during fish production may impact consumer perceptions and opinions of fortified products [17]. Gender and regional variability [25], as well as the sustainability of fish production [26], country of origin, production system, storage conditions, and purchasing price [27], have been identified as the key drivers that affect seafood consumption preferences in Europe. Carlucci et al. [28] performed a systematic review to assess consumer purchasing behavior towards seafood products in the wide context of developed countries and identified the main advantages: the sensory liking of fish, perceived health benefits, and fish-eating habits, while the most important barriers were the sensory disliking of fish, concerns of health risks, perceptions of high price, lack of convenience, lack of availability of preferred fish products, and lack of knowledge in selecting and preparing fish.

Based on this evidence, this study aims to understand how some sensory characteristics of different fish (carp, trout, and seabream) and the presence and absence of information (about the fortification of the fish feed) may impact WTP and how much the consumers like the fish in question. The fortified fish used in this study contain higher levels of iodine, selenium and omega-3 fatty acids compared to conventional fish. These higher levels are obtained during trout farming by adding 3% seaweed to conventional feed, while for the carp and seabream, 2.5% algae, 0.03% selenised yeast, and 6.1% salmon oil were added to the conventional feed. In detail, the study has the following main objective: to understand how the different types of fish (carp, trout, and seabream) and the different fish farming treatments (fortified vs. conventional) impact how much consumers like the product in question and their WTP. In detail, the study aims (i) to figure out if there are any differences in odor, flavor, and taste between the different fortified and conventional fish varieties; (ii) to explore if the information about feed fortification (its presence/absence) impacts how much the consumers like the different types of fish; (iii) to understand whether or not information about feed fortification should be acquired and if it has any impact on the perceived odor, flavor, and taste of the different types of fortified and conventional fish; and (iv) to explore if the evaluation of their WTP during different moments (timing) of the experiment (visual, information, and taste, or visual, taste, and information) could impact the predisposition to pay for the fish. The data were collected as part of a wider investigation at the European level to assess consumer acceptance of eco-innovative seafood solutions and products [29,30].

## 2. Materials and Methods

### 2.1. Participants

A total of 109 participants were recruited through a citizen science initiative (i.e., Le Giornate del Pesce—The fish days, www.giornatedelpesce.org, accessed on 31 August 2020) promoted by a dedicated non-profit network of academic institutions, NGOs, and private companies collaborating with local stakeholders in Piacenza Province, such as fish restaurants and retailers, professional schools for food and catering, and volunteers among students and citizens. An open call for volunteers was promoted via posters and on-line via social networks. A series of public events were organized alongside the sensory analysis (i.e., cooking shows, debates with experts, and practical workshops) to enhance participation and engagement. Only healthy adult volunteers (age range: 18–70 years old) who were living in Italy (no specific information regarding place of birth/residence was requested) and who were responsible for purchasing food and consuming seafood products were included. Vulnerable population groups—specifically pregnant women—and people with relevant food allergies or dietary restrictions (e.g., vegetarians) were excluded from the study. However, only 91 questionnaires were at least half completed and therefore considered valid, with fewer participants answering the questions related to price and liking. The exact number of panelists considered was reported in each statistical analysis result. All the tasters were asked to sign their written informed consent before participating in the study. To guarantee the pseudo-anonymity of the participants and the confidentiality of their data, a code (ID number) was used to identify the participants in the dataset for processing. 

Prior to data collection, protocols were approved by the Medical Ethics Committee of Ghent University Hospital (approval reference number B670201941488) [30], and the research was conducted in accordance with the principles embodied in the Declaration of Helsinki. Data collection was performed according to ICC/ESOMAR principles regarding ethics and standards in social sciences research (ICC/ESOMAR, 2008). 

### 2.2. Fish Samples Obtained with and without Biofortified Feed

Biofortified fish fillets (carp, trout, and seabream), with increased essential nutrients which were developed in the framework of the European project SEAFOODTOMORROW, were submitted for sensory analysis. These fillets were intended to respond to specific consumer needs such as a lack or insufficient ingestion of iodine, selenium, and n-3 fatty acids. Fortified feed was introduced into the fish diets during the final stage (last 2–3 months) of fish farming by incorporating sustainable and natural ingredients rich in iodine (macroalgae), selenium (yeast), and n-3 fatty acids (microalgae or fish by-products) into the feed [29]. Conventional fish fillets (carp, trout, and seabream) farmed in the same conditions as the biofortified fish, minus the incorporation of feed supplementation, represented conventional samples. The fortified fish contained higher levels of iodine, selenium and omega-3 fatty acids compared to conventional ones. These higher levels were obtained during trout farming by adding 3% seaweed to conventional feed, while for the carp and seabream, 2.5% algae, 0.03% selenised yeast, and 6.1% salmon oil were added to conventional feed.

### 2.3. Setting of the Sensory Experiments

**Figure 1:**
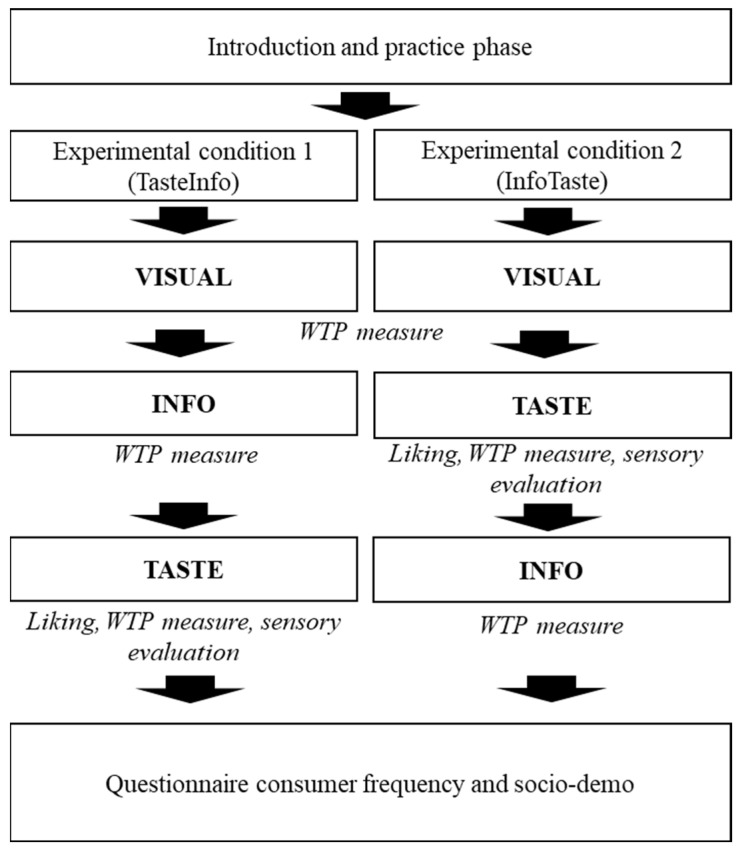
Flowchart of the experiment under two conditions (InfoTaste and TasteInfo). Each participant was randomly assigned to a condition.

The experiments were conducted over three days (27 November and 4 and 5 December 2019) at the SensoryLab of Università Cattolica del Sacro Cuore in Piacenza (Italy) and were fully compliant with ISO standards 8589:2007 “Sensory analysis—General guidance for the design of test rooms”. Each product was served separately on a round white plate labelled with a random three-digit number. Two experimental conditions (TasteInfo and InfoTaste) were performed every day. Participants in the TasteInfo experimental condition were invited to carry out the sensory analysis under blind conditions (i.e., without knowing which of the fish meals were from fish fed biofortified feed). In contrast, participants in the InfoTaste experimental condition performed the sensory analysis after being informed about how the fish products were obtained and about what kind of biofortification was applied. Each participant was randomly assigned to one type of condition (InfoTaste n = 44, TasteInfo n = 47). The panelists rated the pleasantness of the fish varieties on a nine-point scale (1 ‘extremely unpleasant’ to 9 ‘extremely pleasant’) and then stated their WTP during an experimental bid (i.e., the maximum amount of money each participant was willing to pay for a 100 g portion of fish in euros). Participants also evaluated the smell, taste, and color of the cooked fish using a multiple-choice questionnaire, which included 22 adjectives. A simplified scheme presenting the flow of the experiment is proposed below (Figure 1). However, a detailed flow chart of the experiments carried out over the three days, the sensory cards, and the copies of the research instruments used (i.e., questionnaires and protocols) are available in the related project [29,30] and are reported in Supplementary Materials File S1.

### 2.4. Data Analysis

The data were first analyzed for their accuracy of data entry and missing values. The normal variable distribution used in the analysis was evaluated using the skewness and kurtosis indices as the reference values [31]. After that, repeated measure analysis of variance (ANOVA) was conducted to:

- Evaluate how much the consumers liked the three types of fish (sea bream, trout, and carp) by considering the two fish treatments (fortified and conventional) as within-subject measures;
- Evaluate the effects of information availability on “consumer liking” by considering the two experimental conditions: receiving and not receiving information about the nutritional value of the fish, and the two fish treatments (fortified and conventional) as between-subject and within-subject measures, respectively;
- Understand how the information could affect consumer liking compared to the types of fortified fish by analyzing consumer liking of the types of fish (sea bream, trout, and carp) as a within-subject measure as per the two experimental conditions of information disclosure as the between-subject variable;
- Understand how the different fish treatments (fortified and conventional) and the different WTP levels evaluated during the different phases of the experiment (visual, taste, and information phase) could impact the predisposition of the consumers to pay for these products by analyzing the WTP levels and considering the two fish treatments (fortified and conventional) as the within-subject measure.

Bonferroni post hoc analysis was used when ANOVA was significant (*p* < 0.05), and the main effects and interactions were studied at a significance level of *p* = 0.05. Since Mauchly’s test indicated a violation of sphericity, Greenhouse–Geisser correction was used, and ɛ was reported. Additionally, the McNemar test was used to compare the consumers’ perceptions of the appearance, aroma, and taste of the different fish species presented as being conventional or fortified (paired group). Finally, chi-square tests were used to compare the consumers’ sensory perceptions of the different fish varieties when they had or did not have the relevant information on the nutritional properties of the tasted fish (*p* = 0.05). All statistical analyses were performed with SPSS Statistics (Version 20, IBM Corporation, New York, NY, USA).

## 3. Results

The panel of consumers constituted 91 people, 38 (41.8%) of whom were male and 53 (58.2%) of whom were female, aged between 22 and 72 years old (M = 37.59, SD = 14.85). The demographic profile is detailed in Table 1. Moreover, the results show that 46.2% of participants were familiar with carp and consumed it seldomly, 35.2% were familiar with trout and rarely consumed it, and 50.5% of the sample was familiar with seabream but never consumed it.

### 3.1. Impact of the Type of Fish and Feed System (Biofortified and Conventional) on Consumer Liking

Since Mauchly’s test indicated a violation of sphericity, χ^2^(2) = 57.49, *p* < 0.05, the Greenhouse–Geisser correction was used (ɛ = 0.66). We found that the type of fish had the most impact on consumer liking, F(1.311, 102.228) = 131.356, *p* = 0.000, ηp^2^ = 0.627. Bonferroni’s adjusted post-hoc analysis revealed that there were no differences between trout and seabream in terms of pleasantness, while carp was found to be the least pleasant. On the contrary, the type of treatment (fortified vs. conventional) was found to have no significant effect on consumer liking, nor was there any significant interaction between the types of fish presented and the type of treatment (fortified vs. conventional) (Table 2).

### 3.2. Consumer Rating of Appearance, Aroma, and Taste of Fortified and Conventional Samples

When considering the differences perceived by the participants regarding the fortified and conventional trout, a significant difference related to the perception of color was identified. Indeed, most of the participants perceived the fortified trout as being darker and as having a less intense fishy smell than conventional trout.

Regarding the difference in color between the fortified and conventional carp, the majority of the panelists perceived the latter as being pinker in color and having a more intense cooked flavor than the fortified one.

Regarding the differences perceived between conventional and fortified seabream, the majority perceived the fortified seabream as having a more intense cooked taste as well as a stronger oven aroma. All of the differences are detailed in Supplementary Materials File S2.

### 3.3. Impact of Information (Presence/Absence) and Treatment (Fortified and Conventional) on Consumer Liking

A comparison between the results for consumer liking of fortified and conventional fish, depending on whether they had been informed on biofortification before tasting, was carried out. In particular, the two subgroups (those who received the information before tasting and those who were not informed) are comparable to the main socio-demographic features, as they do not differ in terms of gender, age, level of education, and income. As reported in Table 3, significant differences were detected in consumer liking of fortified and conventional fish, as previously reported. However, receiving information (presence vs. absence) had a significant impact on consumer liking (F(1, 77) = 5.918, *p* = 0.017, ηp^2^ = 0.071). A significantly higher “like” was expressed when the sensory analysis was performed after information disclosure. Moreover, there was a significant interaction effect (F(1, 77) = 4.244, *p* = 0.043, ηp^2^ = 0.052) between information status (presence vs. absence) and treatment (fortified vs. conventional). As reported in Table 3, the conventional fish were more appreciated by the consumers who had received information about its nutritional characteristics than by those who did not receive that information, while the consumer’s like of the fortified fish did not change. In short, providing nutritional information about fish increases the consumer’s like of conventional fish, but it does not make a difference for fortified fish.

### 3.4. Impact of Information (Presence/Absence) and Type of Fortified Fish (Trout, Carp, and Seabream) on Consumer Liking

Finally, different types of fortified fish were investigated to understand how this information could affect the perceived liking of each fish species. In particular, the two subgroups (those who received the information before tasting and those who were not informed beforehand) are comparable in terms of the main socio-demographic features, as they do not differ in terms of gender, age, level of education, and income. Since Mauchly’s test indicated a violation of sphericity, χ^2^(2) = 39.92, *p* < 0.05, the Greenhouse–Geisser correction was used (ɛ = 0.72). The results showed that the type of fortified fish had a significant effect on consumer liking, F(1.440, 118.060) = 108.847, *p* = 0.000, ηp^2^ = 0.570. Bonferroni’s adjusted post-hoc analysis revealed that trout and seabream show no differences in terms of pleasantness, while carp is significantly less pleasant. However, information status was found to have no effect on pleasantness (Table 4). Finally, a significant interaction effect (F(2, 164) = 5.461, *p* = 0.005, ηp^2^ = 0.062) was registered between the type of fortified fish and the information provided about fortification before tasting. This fact suggests that the reputation of trout and seabream, already appreciated by Italian consumers because of their sensory characteristics, was not affected by the information about nutritional fortification, as no differences were detected in terms of how much the panelists liked them. In contrast, an increase in knowledge about the nutritional profile of a fish species might improve the appreciation of disliked fish, such as carp.

### 3.5. Consumer Evaluation of Appearance, Aroma, and Taste of Fortified and Conventional Fish, with the Absence or Presence of Information

Comparing the scores for the sensory attributes of the different fish species shows that the fortified or conventional fish were perceived differently by the consumers depending on whether they received information about what they were tasting (see Supplementary Materials File S3). Particularly, regarding fortified trout, among those who received the information, people did not feel as positively about the taste of the cooked fish (X^2^(1) = 3.986, *p* = 0.046) and associated it with fattiness (X^2^(1) = 7.243, *p* = 0.007) when compared to those who did not receive the information. Considering conventional trout, those who did receive the information perceived less of a milky smell (X^2^(1) = 6.812, *p* = 0.009) as well as a less acidic taste (X^2^(1) = 3.917, *p* = 0.048) than the people who did receive the information.

Concerning conventional carp, the consumers who received the information perceived this fish as having a more intense fishy odor (X^2^(1) = 5.022, *p* = 0.025) but a less intense milky odor (X^2^(1) = 16.778, *p* = 0.000), a less acidic odor (X^2^(1) = 11.418, *p* = 0.001), and an acidic flavor (X^2^(1) = 5.557, *p* = 0.018). They also perceived conventional carp as having a lower oven aroma (X^2^(1) = 4.561, *p* = 0.033) and being less fatty (X^2^(1) = 7.350, *p* = 0.007). Regarding fortified carp, those who received the information perceived it as having less of a milky odor (X^2^(1) = 7.350, *p* = 0.000) and a less acidic odor (X^2^(1) = 17.853, *p* = 0.000) but a higher muddy aroma (X^2^(1) = 19.185, *p* = 0.000). Regarding taste, those who received the information perceived the fortified carp as having a very intense muddy taste (X^2^(1) = 14.765, *p* = 0.000) and less of a fishy taste (X^2^(1) = 9.153, *p* = 0.002).

Considering conventional seabream, the consumers who received the information perceived it as being paler in color than those who did not (X^2^(1) = 4.669, *p* = 0.031) and as having a less dark color (X^2^(1) = 6.601, *p* = 0.010) and a more intense fishy (X^2^(1) = 38.386, *p* = 0.000) and cooked (X^2^(1) = 23.968, *p* = 0.000) odor and a less intense milky (X^2^(1) = 25.772, *p* = 0.000) and acidic (X^2^(1) = 47.138, *p* = 0.000) odor. As for taste, those who received the information perceived it as having a greater fishy aftertaste (X^2^(1) = 58.723, *p* = 0.000), as being less acidic (X^2^(1) = 8.211, *p* = 0.004), and as having a less intense milky aftertaste (X^2^(1) = 14.446, *p* = 0.000). Finally, the presence/absence of the information also influenced the perception of the number of fish bones, as those who did not receive the relevant information perceived the product as having fewer bones than those who did receive the information (X^2^(1) = 5.162, *p* = 0.023). Finally, for the fortified seabream, those consumers who received the information perceived it as having a more intense fishy (X^2^(1) = 22.310, *p* = 0.000) and cooked (X^2^(1) = 14.765, *p* = 0.000) odor but a less intense milky (X^2^(1) = 10.777, *p* = 0.001) and acidic (X^2^(1) = 63.894, *p* = 0.000) odor compared to those who did not. Finally, as far as taste was concerned, the consumers who received the information perceived a more intense fishy aftertaste (X^2^(1) = 55.306, *p* = 0.000) and a less intense oven aroma aftertaste (X^2^(1) = 7.027, *p* = 0.008).

### 3.6. Impact of Fish Type (Carp, Trout, and Seabream) and Treatment (Fortified/Conventional) on WTP

Since Mauchly’s test indicated a violation of sphericity, χ^2^(2) = 18.863, *p* < 0.0001, Greenhouse–Geisser correction was used (ɛ = 0.84). The results showed (Table 5) that the type of fish (carp, trout, and seabream) had a significant effect on consumers’ WTP, F(1.674, 147.290) = 76.403, *p* = 0.000, ηp^2^ =0.465. Bonferroni’s adjusted post-hoc analysis revealed that there were no differences between trout and seabream in terms of WTP, while the consumers were predisposed to pay less for carp. Moreover, the type of treatment (fortified vs. conventional) was observed to have a significant effect on consumer WTP, F(1, 88) = 13.121 *p* = 0.000, ηp^2^ = 0.130. Consumers are predisposed to pay more for fortified fish than for their conventional counterparts. No interaction effect was observed between types of fish and fish treatment (fortified vs. conventional). However, when the WTP for the same type of conventional and fortified fish is considered, people were predisposed to pay more for fortified seabream than for the conventional variety.

### 3.7. Impact of the Timing of WTP Evaluation and Fish Farming Treatment (Fortified/Conventional) on the WTP

To conclude, the data show that the treatment (fortified vs. conventional) and the time at which WTP was determined (evaluated during the visual, information, and tasting phase in the first experimental condition and during the visual, tasting, and information phase in the second experimental condition) impact this parameter (Table 6). Considering the first experimental condition (in which the WTP was assessed during the visual–info–taste phase), this study shows that different times of detection have an effect on consumers’ willingness to pay (evaluated during the visual, information, and tasting phases): F(2, 84) = 5.868, *p* = 0.004, and ηp^2^ = 0.123. Bonferroni’s adjusted post-hoc test revealed that during tasting, people are willing to pay less on average than during the visual and information phases. In addition, we noted that fish treatment (fortified vs. conventional) also had an impact on WTP (F(1, 42) = 5.847, *p* = 0.020, ηp^2^ = 0.122). Specifically, people are willing to pay more for fortified fish than for conventional fish, as reported in previous analyses. In addition, there is an interaction effect between the timing of detection of WTP (evaluated during the visual, information and tasting phase) and the type of fish treatment (fortified or conventional) (F(2, 84) = 8.766, *p* = 0.000, ηp^2^ = 0.173). Moreover, the phase in which information on both fish treatment (fortified or conventional) was given caused a significant increase in WTP for fortified fish compared to the visual phase, while WTP related to conventional fish did not change significantly. It is also interesting to note that after tasting the fortified fish, the WTP for it decreased dramatically.

Lastly, the second experimental condition (in which WTP was assessed during the visual, taste, and information phase), the effect of fish treatment (fortified vs. conventional) was identified for WTP (F(1, 45) = 7. 435, *p* = 0.009, ηp^2^ = 0.142) (Table 7). Consumers are willing to pay more for fortified fish than for conventional fish, as was also found in the first experimental condition. An effect of different detection time on WTP (evaluated during the visual, tasting, and information phase) was observed (F(1551, 69,817) = 13.131, *p* = 0.000, ηp^2^ = 0.226). Bonferroni-adjusted post-hoc analysis revealed that, during the tasting, consumers are, on average, willing to pay less, while they are willing to pay more during the visual phase. There was no interaction effect between detection time and fish treatment (fortified vs. conventional).

## 4. Discussion

Among the different fish species tested in this study, carp (both fortified and conventional) was less appreciated by the participants, while seabream and trout were more liked. This can be explained through the concept of familiarity [32,33,34], which explains how people tend to prefer known products that they buy often. Indeed, carp is a fish that is rarely eaten and purchased in Italy [35]. On the contrary, no major differences were found in pleasantness between fortified and conventional fish, regardless of species. This is relevant since, for other types of consumption (for example, enriched foods), making the product healthier and more sustainable is a factor that often results in increased consumer liking [36,37]. Moreover, the sensory evaluation of the appearance, taste, and odor of the three fortified and conventional fish species enabled us to confirm that consumers can perceive differences among them, even if these distinctions do not impact judgements about pleasantness. Additionally, these results do not distinguish between those who received/did not receive treatment information before tasting the fish. Overall, this study highlights that when participants have information about what they are eating beforehand, it leads them to perceive fish as being tastier than they would without prior information. When considering the interaction between the type of fish treatment (fortified vs. conventional) and the information provided, the level of like of the fortified fish was not affected, whereas the conventional fish were preferred when the information was available. Consequently, the information related to fish (conventional/fortified) did not enhance the perceived value of the fortified fish but increased the consumer liking of the conventional fish. This might also be due to the way in which the information was given since how a product is communicated has a substantial impact on consumer choices, especially those concerning food [38,39,40,41].

In terms of how the absence or the presence of information against the three different fortified fish species impacted consumer liking, carp was less appreciated, while no differences between seabream and trout were highlighted. It is noteworthy to mention that, among the fortified fish, information about what is being tasted leads to an increase in the liking of fortified carp, but this was not the case for the other fish varieties. This finding can only be explained for the less liked products, i.e., carp, which received very low liking scores compared to other fish, indicating that fortification plays the role of a taste enhancer. Since seabream and trout are commonly appreciated by consumers, being fortified was not recognized as resulting in a meaningful sensory improvement. On the contrary, fortification enables us to valorize carp, thus impacting perceptions of consumer liking; that is to say (unconsciously), “I do not like it, but at least it is fortified”. These results are significant because they allow us to understand that the impact of fortification on consumer liking changes according to fish species. Similar results justifying this evidence can be found in previous studies on the liking and acceptance of bitter vegetables [42], where it was shown how the same informative labels have a different impact when applied to different products and how this depends on food features [43,44].

Regarding WTP, the consumers were predisposed to pay more for seabream and trout than for carp. In addition, WTP was greater for fortified fish than for conventional fish. Finally, the treatment (fortified vs. conventional) and when WTP was detected, interacted, and had an impact on consumers’ WTP. Consumers participating in the first experimental session, when WTP towards fortified and conventional fish was determined in three different moments (in this order: after seeing the fish, after receiving the information, and after having an informed taste), were willing to pay slightly more for the fortified fish, especially just after having received the information. This highlights how being aware of the fortified status of a fish leads to a greater predisposition to pay more. However, when a fortified fish was tasted after the participants had received the relevant information, a drastic decrease in the predisposition to pay was highlighted, and the WTP level went back to values like those declared for conventional fish. This was probably because consumers’ expectations for fortified fish were disillusioned during and after tasting, as already shown in past studies [45]. Additionally, in this case, it was interesting to evaluate the meaning and expectation that the presence of information had on the consumer. In the case of liking, it is important to create communication messages that do not disillusion consumer expectations, which, in turn, leads to a lower predisposition to pay more.

Some important limitations of this research need to be addressed in the design of future studies. The first limitation relates to the representativeness of the sample across the quantitative exploratory data collection performed: indeed, only healthy adult volunteers, ranging between 18 and 70 years of age, were included in the research, excluding a relevant portion of the entire population (e.g., about 17% of the Italian population is above 70). Moreover, the sample comprised a high proportion of people under 34 years old as well as more highly educated individuals, probably due to the recruitment modality (i.e., citizen science activities organized in academic facilities). Hence, the results should be interpreted with care, and further research should try to reach individuals with an age and education level representative of the Italian population level.

Moreover, previous research [25,46] suggested that fish consumers can be clustered into three distinct groups based on general health interests, the perceived benefits of eating seafood, and attitudes towards seafood (i.e., health seekers who eat seafood for duty; health seekers and seafood lovers; low commitment to health and indifference to seafood), and further research should aim to obtain a detailed analysis of pleasantness and WTP and their relationship within different consumer groups. Without considering these target groups, this study can provide interesting but not optimal and timely insights into groups of consumers. Another limitation of this research relies on, as with almost all studies on consumers’ WTP, the gap between the stated and revealed or real WTP. This so-called attitude-behavior gap is frequently cited when observing consumers stating a high WTP in surveys but who behave quite differently in the marketplace [47]. However, consumers’ WTP in the marketplace depends on the way that specific product attributes are communicated and on the credibility of the communicator and the content to a very large degree. 

## 5. Conclusions

To maximize consumer satisfaction and the future purchase of fortified fish, it is important to understand consumer preferences and their WTP for these products. The results confirmed how fortification acquires a value for consumers, especially for fish which are commonly liked and if information with respect to fortification is available. However, it is important to reconsider how information impacts consumer expectations, as when it was supplied during the tasting phase, it made the participant’s WTP dramatically drop. Further studies on communication frameworks that are useful for understanding the most impactful topics regarding product acceptability are recommended. Finally, these findings evidence that consumers are partially relying on erroneous information about fortified products when deciding to purchase fish. Hence, encouraging an open platform between experts and the public to discuss and debate the ethical, environmental, and safety issues surrounding fish breeding using biofortified feed might raise consciousness and provide scientific information and facts for consumers to make informed decisions.

## Figures and Tables

**Table 1:** Demographic profiles of the sample of participants (n = 91).

Gender	n	%
Male	38	41.8
Female	53	58.2
**Age**		
18–24	24	26.4
25–34	24	26.4
35–44	13	14.2
45–54	14	15.4
55–59	5	5.5
60–72	11	12.1
**Education**		
Primary or secondary	27	29.7
Higher education	64	70.3
**Geographic area**		
Suburb or hinterland of a large city	6	6.6
Countryside	17	18.7
Small town	57	62.6
Big city	11	12.1
**Profession**		
Paid work (24 h per week or more)	34	37.4
Housewife	2	2.2
Student	30	33.0
Retired	8	8.8

**Table 2:** Overall liking of fortified and conventional fish according to fish type ^1^.

	Trout		Carp		Seabream		Average Liking Score	
	M	SD	M	SD	M	SD	M	SD
Fortified	6.82 ^b,A^	1.37	4.05 ^a,A^	2.33	7.13 ^b,A^	1.28	6.00 ^A^	1.66
Conventional	6.95 ^b,A^	1.28	4.25 ^a,A^	2.34	7.15 ^b,A^	1.11	6.11 ^A^	1.57
Average liking score	6.88 ^b^	1.32	4.15 ^a^	2.33	7.14 ^b^	1.19		

^1^ n = 79; M = mean; SD = standard deviation; Different lowercase and uppercase letters identify significant differences between means in columns and in rows, respectively.

**Table 3:** Overall liking of fortified and conventional fish according to the rate of acquired information before sensory evaluation ^1^.

	Fortified		Conventional		Average Liking Score	
	M	SD	M	SD	M	SD
InfoTaste (n = 37)	6.18 ^a,A^	1.37	6.53 ^b,A^	0.96	6.35 ^A^	1.16
TasteInfo (n = 42)	5.84 ^a,A^	0.95	5.75 ^a,B^	1.16	5.79 ^B^	1.05
Average liking score	6.00 ^a^	1.17	6.14 ^a^	1.13		

^1^ n = 79; M = mean; SD = standard deviation; different lowercase and uppercase letters identify significant differences between means in columns and in rows, respectively.

**Table 4:** Overall liking of fortified fish according to the rate of acquired information before sensory evaluation ^1^.

	Fortified Trout		Fortified Carp		Fortified Seabream		Average Liking Score	
	M	SD	M	SD	M	SD	M	SD
InfoTaste (n = 38)	7.05 ^a^^,A^	1.41	4.55 ^b^^,A^	2.39	6.95 ^a^^,A^	1.54	6.18 ^A^	1.78
TasteInfo (n = 46)	6.57 ^a^^,A^	1.33	3.37 ^b^^,B^	2.20	7.33 ^a^^,A^	0.97	5.75 ^A^	1.50
Average liking score	6.81 ^a^	1.37	3.96 ^b^	2.30	7.14 ^a^	1.25		

^1^ n = 84; M = mean; SD = standard deviation; different lowercase and uppercase letters identify significant differences between means in columns and in rows, respectively.

**Table 5:** Overall WTP for fortified/conventional fish and types of fish ^1^.

	Trout		Carp		Seabream		Average WTP Score	
	M	SD	M	SD	M	SD	M	SD
Fortified	EUR 13.93 ^b,A^	4.46	EUR 8.11 ^a,B^	4.80	EUR 15.07 ^b,A^	5.34	EUR 12.37 ^A^	4.87
Conventional	EUR 13.43 ^b,A^	4.43	EUR 7.89 ^a,B^	4.70	EUR 14.07 ^b,B^	5.46	EUR 11.94 ^B^	4.86
Average WTP score	EUR 13.68 ^a^	4.45	EUR 8.00 ^b^	4.75	EUR 14.79 ^a^	5.40		

^1^ n = 89; M = mean; SD = standard deviation; different lowercase and uppercase letters identify significant differences between means in columns and in rows, respectively.

**Table 6:** Overall WTP for fortified/conventional fish according to the stage of detection in the first experimental condition (visual, information, and taste) ^1^.

	Visual		Info		Taste		Average WTP Score	
	M	SD	M	SD	M	SD	M	SD
Fortified	EUR 12.01 ^b,A^	4.29	EUR 13.20 ^a,A^	4.58	EUR 11.48 ^b,A^	4.19	EUR 12.24 ^A^	4.35
Conventional	EUR 12.40 ^a,A^	4.41	EUR 11.82 ^a,b,B^	4.10	EUR 11.47 ^b,A^	3.96	EUR 11.90 ^B^	4.16
Average WTP score	EUR 12.21 ^a,b^	4.35	EUR 12.52 ^a^	4.34	EUR 11.48 ^b^	4.07		

^1^ n = 43; M = mean; SD = standard deviation; different lowercase and uppercase letters identify significant differences between means in column and in row, respectively.

**Table 7:** Overall WTP for fortified/conventional fish according to the stage of detection in the second experimental condition (visual, taste, and information) ^1^.

	Visual		Taste		Info		Average WTP Score	
	M	SD	M	SD	M	SD	M	SD
Fortified	EUR 13.31 ^b,A^	3.41	EUR 11.56 ^a,A^	3.16	EUR 12.60 ^b,A^	3.62	EUR 12.50 ^A^	3.40
Conventional	EUR 12.99 ^a,A^	3.52	EUR 11.35 ^b,A^	3.61	EUR 11.58 ^b,B^	3.23	EUR 11.98 ^B^	3.45
Average WTP score	EUR 13.16 ^a^	3.47	EUR 11.46 ^b^	3.39	EUR 12.09 ^c^	3.42		

^1^ n = 46; M = mean; SD = standard deviation; different lowercase and uppercase letters identify significant differences between means in columns and in rows, respectively.

## Data Availability

Research data are available in the article’s Supplementary Materials and upon request from the authors.

## Supplementary Materials

The following supporting information can be downloaded at: https://www.mdpi.com/article/10.3390/foods11182808/s1, File S1: Flow of the experimental process during sensory evaluation; File S2: Consumer ratings of appearance, aroma, and taste of fortified and conventional samples; File S3: Consumer evaluations of appearance, aroma, and taste of fortified and conventional fish with the absence or presence of information.
