# Supplementary material for: Perceptions and Liking Distortion from Information about the Nutritional Upgrades in Biofortified Seafood Products
Source: Foods. 2022 Sep 12;11(18):2808. doi: 10.3390/foods11182808 (PMC9497835; doi:10.3390/foods11182808)
Supplement: Supplementary file 1 [file foods-11-02808-s001.zip › File S1.pdf]

Flow of the experimental auctions with sensory evaluation Session A-D

Sequence	Treatment 1 InfoTaste			Treatment 2 TasteInfo		
	Session A			Session D		
1	Instruction and practice round			Instruction and practice round		
2	Hunger level assessment			Hunger level assessment		
	Randomise			*Randomise*		
	**Trout**	**Carp**	**Seabream**	**Trout**	**Carp**	**Seabream**
3 5 7<br>4 6 8	Visual evaluation<br>Control bid round	Visual evaluation<br>Control bid round	Visual evaluation<br>Control bid round	Visual evaluation<br>Control bid round	Visual evaluation<br>Control bid round	Visual evaluation<br>Control bid round
			E			*E*
9 11 13<br>10 12 14	Info<br>Bid	Info<br>Bid	Info<br>Bid	Taste<br>Bid	Taste<br>Bid	Taste<br>Bid
			A			*C*
15 17 19<br>16 18 20	Taste<br>Bid	Taste<br>Bid	Taste<br>Bid	Info<br>Bid	Info<br>Bid	Info<br>Bid
			B			*D*
21 23 25<br>22 24 26	Reference price<br>Bid	Reference price<br>Bid	Reference price<br>Bid	Reference price<br>Bid	Reference price<br>Bid	Reference price<br>Bid
27 28 29	Draw bidding round for Salmon →	Draw bidding round for Carp →	Draw bidding round for Seabream	Draw bidding round for Salmon →	Draw bidding round for Carp →	Draw bidding round for Seabream
30	Questionnaire consumer frequency and socio-demo			Questionnaire consumer frequency and socio-demo		
31	Each highest bidder for each seafood product purchases the fortified Salmon/Carp/Seabream			Each highest bidder for each seafood product purchases the fortified Salmon/Carp/Seabream		

Flow of the experimental auctions with sensory evaluation Session B-E

Sequence			Treatment 1 InfoTaste			Treatment 2 TasteInfo		
			Session B			Session E		
1			Instruction and practice round			Instruction and practice round		
2			Hunger level assessment			Hunger level assessment		
			Randomise			*Randomise*		
			**Carp**	**Seabream**	**Trout**	**Carp**	**Seabream**	**Trout**
3	5	7	Visual evaluation	Visual evaluation	Visual evaluation	Visual evaluation	Visual evaluation	Visual evaluation
4	6	8	Control bid round	Control bid round	Control bid round	Control bid round	Control bid round	Control bid round
					E			*E*
9	11	13	Info	Info	Info	Taste	Taste	Taste
10	12	14	Bid	Bid	Bid	Bid	Bid	Bid
					A			*C*
15	17	19	Taste	Taste	Taste	Info	Info	Info
16	18	20	Bid	Bid	Bid	Bid	Bid	Bid
					B			*D*
21	23	25	Reference price	Reference price	Reference price	Reference price	Reference price	Reference price
22	24	26	Bid	Bid	Bid	Bid	Bid	Bid
27	28	29	Draw bidding round for Carp	Draw bidding round for Seabream	Draw bidding round for Salmon	Draw bidding round for Carp	Draw bidding round for Seabream	Draw bidding round for Salmon
30			Questionnaire consumer frequency and socio-demo			Questionnaire consumer frequency and socio-demo		
31			Each highest bidder for each seafood product purchases the fortified Carp/Seabream/Salmon			Each highest bidder for each seafood product purchases the fortified Carp/Seabream/Salmon		

Flow of the experimental auctions with sensory evaluation Session C-F

Sequence			Treatment 1 InfoTaste			Treatment 2 TasteInfo		
			Session C			Session F		
1			Instruction and practice round			Instruction and practice round		
2			Hunger level assessment			Hunger level assessment		
			Randomise			*Randomise*		
			**Seabream**	**Trout**	**Carp**	**Seabream**	**Trout**	**Carp**
3	5	7	Visual evaluation	Visual evaluation	Visual evaluation	Visual evaluation	Visual evaluation	Visual evaluation
4	6	8	Control bid round	Control bid round	Control bid round	Control bid round	Control bid round	Control bid round
					E			*E*
9	11	13	Info	Info	Info	Taste	Taste	Taste
10	12	14	Bid	Bid	Bid	Bid	Bid	Bid
					A			*C*
15	17	19	Taste	Taste	Taste	Info	Info	Info
16	18	20	Bid	Bid	Bid	Bid	Bid	Bid
					B			*D*
21	23	25	Reference price	Reference price	Reference price	Reference price	Reference price	Reference price
22	24	26	Bid	Bid	Bid	Bid	Bid	Bid
27	28	29	Draw bidding round for Seabream	Draw bidding round for Salmon	Draw bidding round for Carp	Draw bidding round for Seabream	Draw bidding round for Salmon	Draw bidding round for Carp
30			Questionnaire consumer frequency and socio-demo			Questionnaire consumer frequency and socio-demo		
31			Each highest bidder for each seafood product purchases the fortified Seabream/Salmon/Carp			Each highest bidder for each seafood product purchases the fortified Seabream/Salmon/Carp		

SEAFOOD TOMORROW

WELCOME to this experiment!

UGent/CBHU/Aeiforia

Researchers: (fill-in the names of the involved researchers at your institution)

This project has received funding from the European Union's Horizon 2020 research and innovation programme under Grant Agreement no. 773400 (SEAFOODTOMORROW). This output reflects the views only of the author(s), and the European Union cannot be held responsible for any use which may be made of the information contained therein.

Instructions

SEAFOOD TOMORROW

- Thank you for choosing to participate in our experiment on how people make food choices.
- Please read carefully all the instructions that will appear on the screen.
- There is **no right or wrong answer** in any question, we just want to know your opinion.
- Because we are trying to find out the value for the food products, it is very important **not to communicate with any other participant** for any reason. Any attempt to communicate with each other will result in the failure of this experiment.

This project has received funding from the European Union's Horizon 2020 research and innovation programme under Grant Agreement no. 773400 (SEAFOOD[TOMORROW]). This output reflects the views only of the author(s), and the European Union cannot be held responsible for any use which may be made of the information contained therein.

Instructions

- Today we will auction some seafood products. In a while you will receive details on how an auction works.
- Your participation in the experiment will be rewarded with the amount of **€ __.** This money is yours to use as you wish. We will also give you the opportunity to buy some food products during the auctions that are about to follow.
- This study is **not hypothetical and not just a game**, you should know that if you are the **highest bidder** in an auction you will **have to pay for the product** you bid.

This project has received funding from the European Union's Horizon 2020 research and innovation programme under Grant Agreement no. 773400 (SEAFOOD[TOMORROW]). This output reflects the views only of the author(s), and the European Union cannot be held responsible for any use which may be made of the information contained therein.

How does the auction work?

- **Step 1.** You will first examine the food products we are about to auction.
- **Step 2.** Each one of you, will submit a bid for each food product in the appropriate field on the paper.
- **Step 3.** We will rank bids from the highest to the lowest.
- **Step 4.** The person that submits the highest bid wins the auction but will pay the price of the second highest bidder. In case of ties, the highest bidder would be selected randomly.

This project has received funding from the European Union's Horizon 2020 research and innovation programme under Grant Agreement no. 773400 (SEAFOODTOMORROW). This output reflects the views only of the author(s), and the European Union cannot be held responsible for any use which may be made of the information contained therein.

How does the auction work?

- In this type of auction, it is always in your best interest to submit **bids exactly equal to your valuation for the product**. If you submit a bid **higher** than your valuation for the product, the second highest price may be higher than what you are willing to pay but **you will still need to pay for it**.
- In addition, you do not gain by submitting bids **lower** than your true willingness to pay. This is because the highest bidder generally **does not pay the amount he/she offers**, but the price submitted by the second highest bidder. Besides, this also **reduces your chance** of obtaining the product. Therefore, you should always **submit bids that are exactly equal to the price you want to pay for the product.**

This project has received funding from the European Union's Horizon 2020 research and innovation programme under Grant Agreement no. 773400 (SEAFOOD TOMORROW). This output reflects the views only of the author(s), and the European Union cannot be held responsible for any use which may be made of the information contained therein.

Visual evaluation

Visual evaluation

ID ________ Groep ___

Bid Sheet

Visual evaluation

The most I am willing to pay for …

… **Trout 842** is ______________€

… **Trout 199** is ______________€

This project has received funding from the European Union's Horizon 2020 research and innovation programme under Grant Agreement no. 773400 (SEAFOODTOMORROW). This output reflects the views only of the author(s), and the European Union cannot be held responsible for any use which may be made of the information contained therein.

Visual evaluation

ID ________ Groep ___

Bid Sheet

Visual evaluation

The most I am willing to pay for …

… **Carp 524** is ______________€

… **Carp 246** is ______________€

This project has received funding from the European Union's Horizon 2020 research and innovation programme under Grant Agreement no. 773400 (SEAFOODTOMORROW). This output reflects the views only of the author(s), and the European Union cannot be held responsible for any use which may be made of the information contained therein.

Visual evaluation

SEAFOOD TOMORROW

ID ________ Groep ___

Bid Sheet

Visual evaluation

The most I am willing to pay for …

… **Seabream 902** is ______________€

… **Seabream 734** is ______________€

This project has received funding from the European Union's Horizon 2020 research and innovation programme under Grant Agreement no. 773400 (SEAFOOD TOMORROW). This output reflects the views only of the author(s), and the European Union cannot be held responsible for any use which may be made of the information contained therein.

Information

Information

- Trout

The differences between the two products are as follows:

Trout 842 is the **conventional trout fillet** that you can find in the market. **Trout 199** is a **biofortified Trout** that contains **higher levels of Iodine and Selenium** compared to trout 842. Theses higher levels are obtained during production of the trout by adding 3% seaweed to conventional feed to enrich Iodine and Selenium levels in the seafood products. This biofortified trout has the goal **to improve and to respond better to specific needs of the consumers.**

This project has received funding from the European Union's Horizon 2020 research and innovation programme under Grant Agreement no. 773400 (SEAFOODTOMORROW). This output reflects the views only of the author(s), and the European Union cannot be held responsible for any use which may be made of the information contained therein.

Information

ID ________ Groep ___

Bid Sheet

Information

The most I am willing to pay for …

… **Trout 842** is ______________€

… **Trout 199** is ______________€

This project has received funding from the European Union's Horizon 2020 research and innovation programme under Grant Agreement no. 773400 (SEAFOOD TOMORROW). This output reflects the views only of the author(s), and the European Union cannot be held responsible for any use which may be made of the information contained therein.

Information

- **Carp**

The differences between the two products are as follows:

Carp 246 is the **conventional carp fillet** that you can find in the market. **Carp 524** is a **biofortified carp** that contains **higher levels of Iodine, Selenium and Omega-3 fatty acids** compared to carp 246. Theses higher levels are obtained during production of the carp by adding 0,5% algae, 0.03% selenised yeast, 6.1 salmon oil to conventional feed to enrich Iodine, Selenium and Omega-3 fatty acids levels in the seafood products. This biofortified carp has the goal **to improve and to respond better to specific needs of the consumers.**

This project has received funding from the European Union's Horizon 2020 research and innovation programme under Grant Agreement no. 773400 (SEAFOODTOMORROW). This output reflects the views only of the author(s), and the European Union cannot be held responsible for any use which may be made of the information contained therein.

Information

ID ________ Groep ___

Bid Sheet

Information

The most I am willing to pay for …

… **Carp 524**is _______________€

… **Carp 246** is _______________€

This project has received funding from the European Union's Horizon 2020 research and innovation programme under Grant Agreement no. 773400 (SEAFOOD TOMORROW). This output reflects the views only of the author(s), and the European Union cannot be held responsible for any use which may be made of the information contained therein.

Information

- **Seabream**

The differences between the two products are as follows:

Seabream 902 is the **conventional seabream** that you can find in the market. **Seabream 734** is a **biofortified seabream** that contains **higher levels of Iodine and Selenium** compared to seabream 902. Theses higher levels are obtained during production of the seabream by adding 0,5% macroalgae and 0,03% selenised-yeast to the feed to enrich Iodine and Selenium levels in the seafood products. This biofortified seabream has the goal **to improve and to respond better to specific needs of the consumers.**

This project has received funding from the European Union's Horizon 2020 research and innovation programme under Grant Agreement no. 773400 (SEAFOOD[TOMORROW]). This output reflects the views only of the author(s), and the European Union cannot be held responsible for any use which may be made of the information contained therein.

Information

ID ________ Groep ___

Bid Sheet

Information

The most I am willing to pay for …

… **Seabream 902**is ______________€

… **Seabream 734** is ______________€

This project has received funding from the European Union's Horizon 2020 research and innovation programme under Grant Agreement no. 773400 (SEAFOODTOMORROW). This output reflects the views only of the author(s), and the European Union cannot be held responsible for any use which may be made of the information contained therein.

Sensory evaluation

Sensory evaluation

ID ________ Groep ___

Bid Sheet

Sensory evaluation

The most I am willing to pay for …

… **Trout 842**is ______________€

… **Trout 199** is ______________€

This project has received funding from the European Union's Horizon 2020 research and innovation programme under Grant Agreement no. 773400 (SEAFOODTOMORROW). This output reflects the views only of the author(s), and the European Union cannot be held responsible for any use which may be made of the information contained therein.

Sensory evaluation

ID ________ Groep ___

Bid Sheet

Sensory evaluation

The most I am willing to pay for …

… **Carp 524**is ______________€

… **Carp 246** is ______________€

This project has received funding from the European Union's Horizon 2020 research and innovation programme under Grant Agreement no. 773400 (SEAFOOD TOMORROW). This output reflects the views only of the author(s), and the European Union cannot be held responsible for any use which may be made of the information contained therein.

Sensory evaluation

ID ________ Groep ___

Bid Sheet

Sensory evaluation

The most I am willing to pay for …

… **Seabream 902**is ______________€

… **Seabream 734** is ______________€

This project has received funding from the European Union's Horizon 2020 research and innovation programme under Grant Agreement no. 773400 (SEAFOOD TOMORROW). This output reflects the views only of the author(s), and the European Union cannot be held responsible for any use which may be made of the information contained therein.

Thank you for your participation!

E-mail address & contact info main researcher
