# Supplementary material for: Perceptions and Liking Distortion from Information about the Nutritional Upgrades in Biofortified Seafood Products
Source: Foods. 2022 Sep 12;11(18):2808. doi: 10.3390/foods11182808 (PMC9497835; doi:10.3390/foods11182808)
Supplement: Supplementary file 1 [file foods-11-02808-s001.zip › File S2.pdf]

Supplementary Material File S2. Consumer rating of appearance, aroma, and taste of fortified and conventional samples

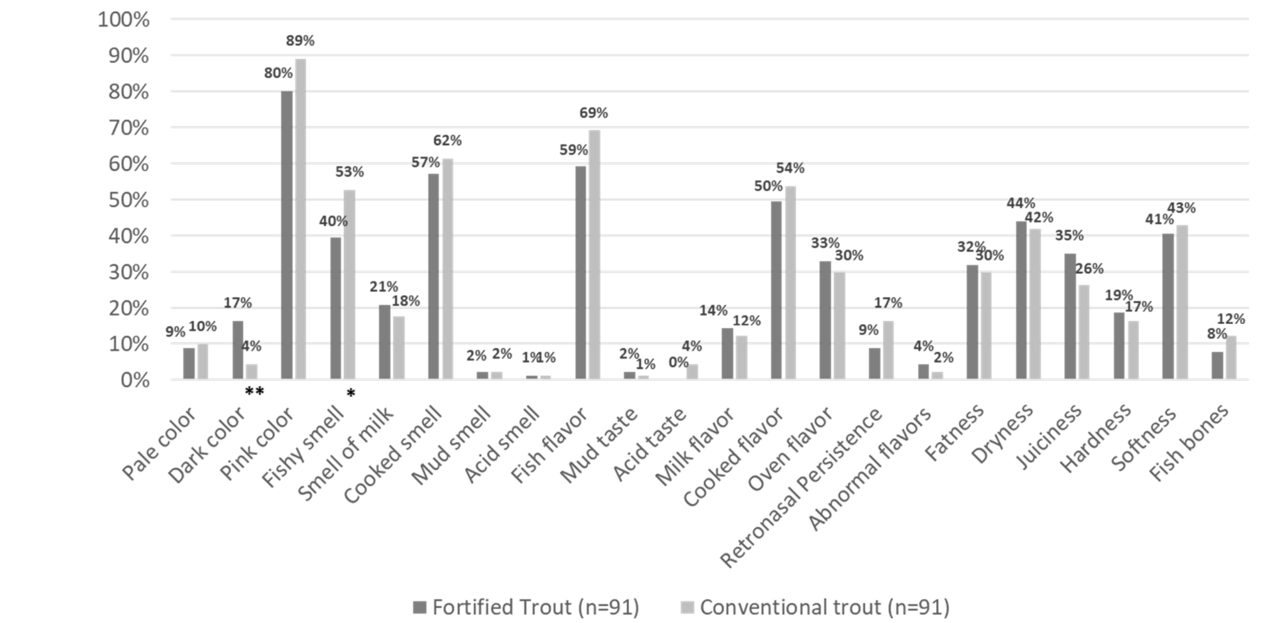

Level of significance: **p < 0.01, *p<0.05 - McNemar test

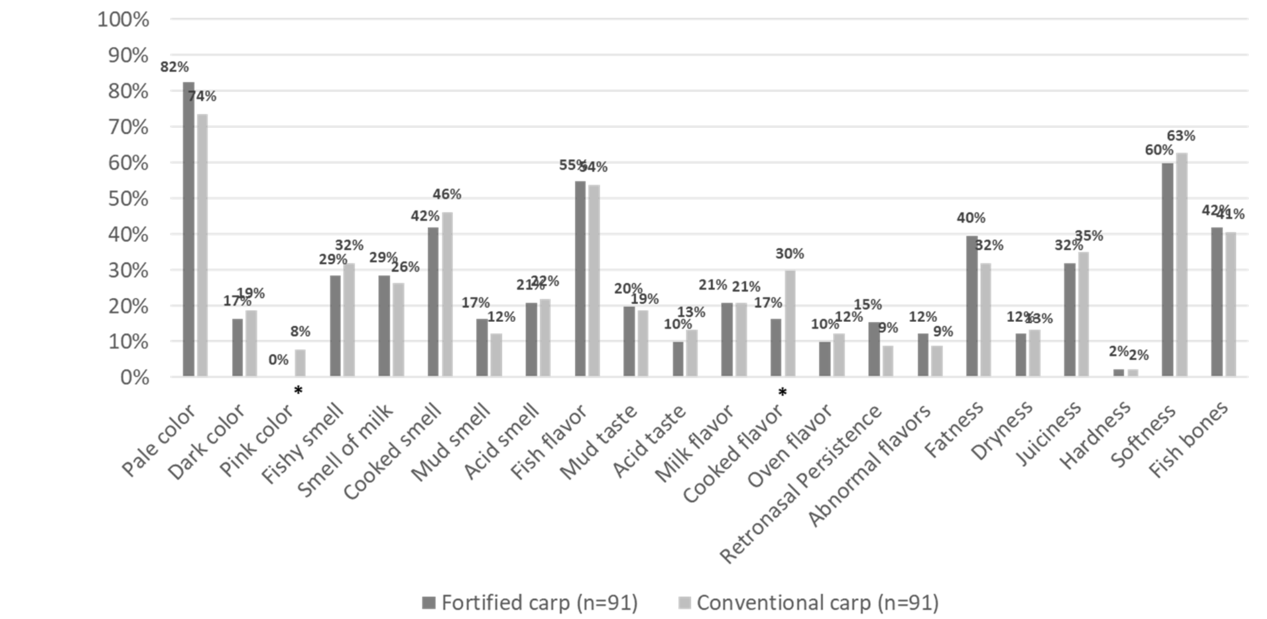

Level of significance: **p < 0.01, *p<0.05 - McNemar test

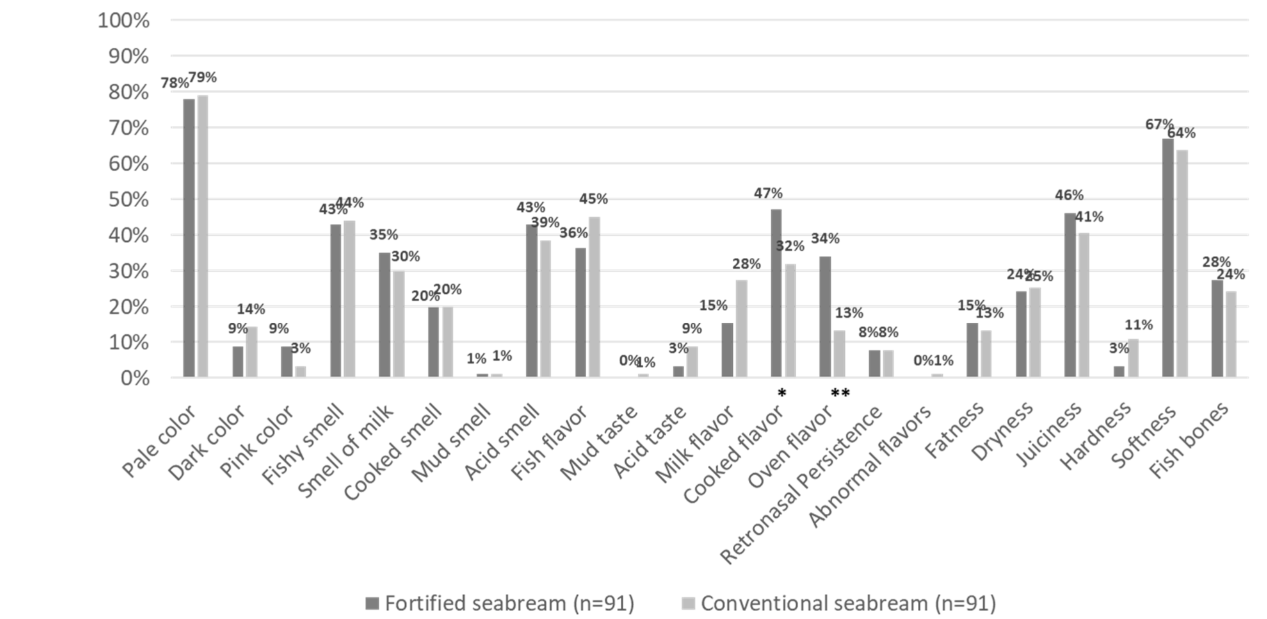

Level of significance: **p < 0.01, *p<0.05 - McNemar test
