# Supplementary material for: Perceptions and Liking Distortion from Information about the Nutritional Upgrades in Biofortified Seafood Products
Source: Foods. 2022 Sep 12;11(18):2808. doi: 10.3390/foods11182808 (PMC9497835; doi:10.3390/foods11182808)

Supplementary Material File S3. Consumer evaluation of appearance, aroma, and taste of fortified and conventional fish with the absence or presence of information

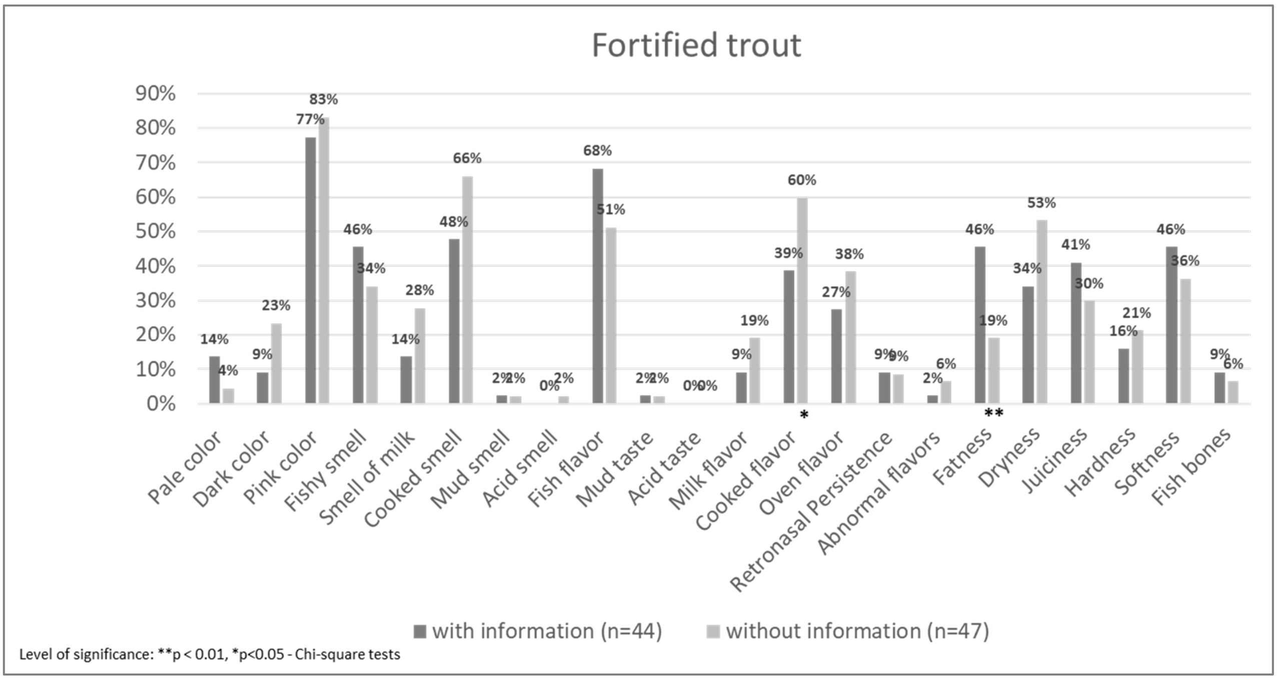

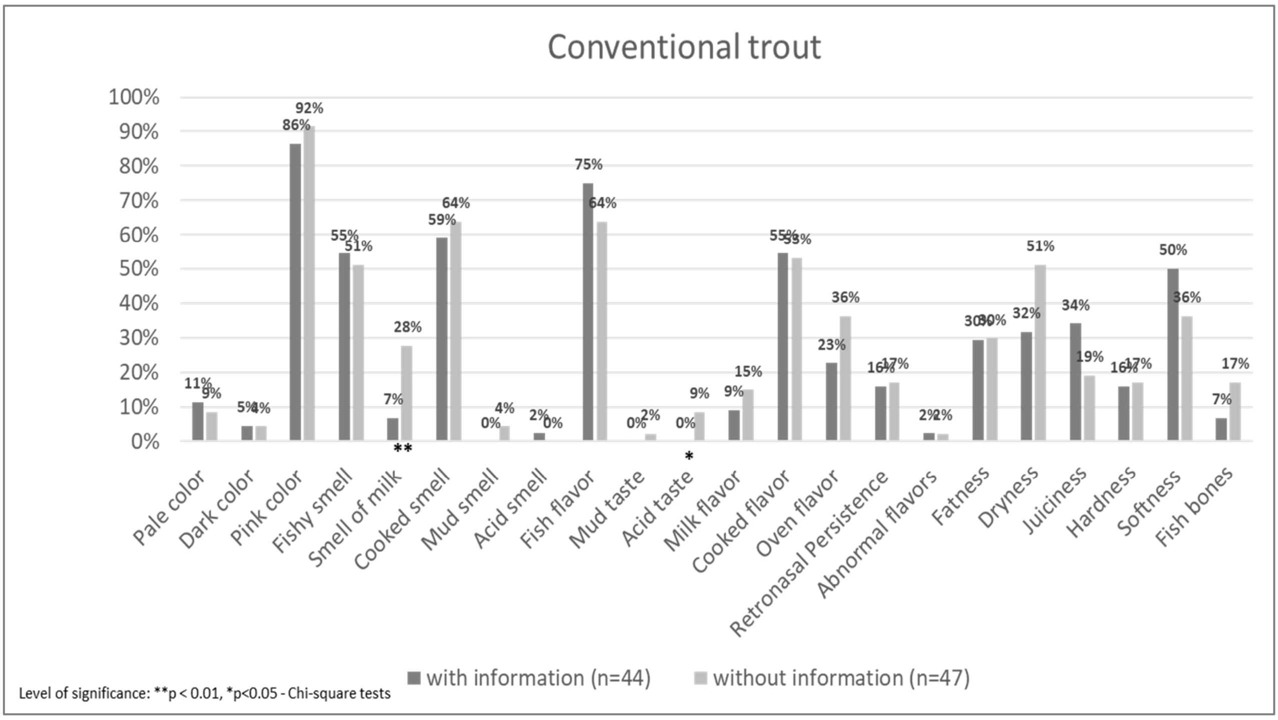

Supplementary Material File S3. Consumer evaluation of appearance, aroma, and taste of fortified and conventional fish with the absence or presence of information

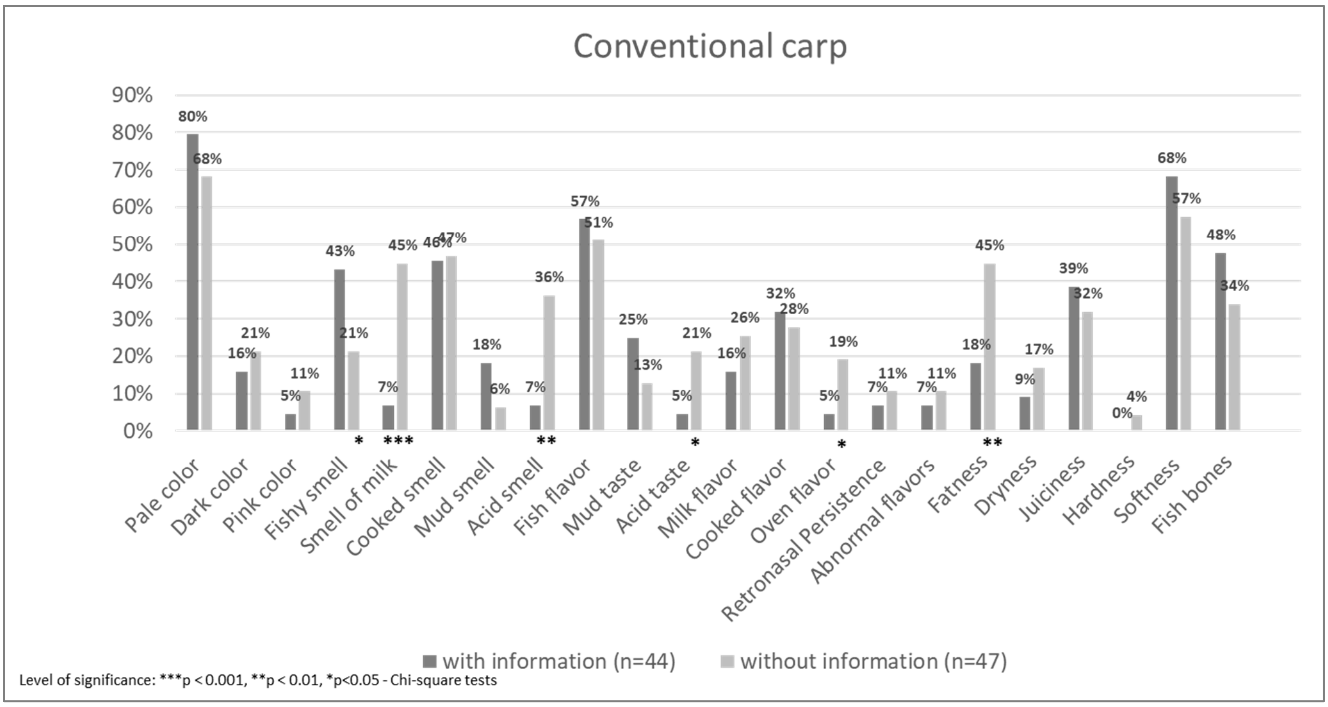

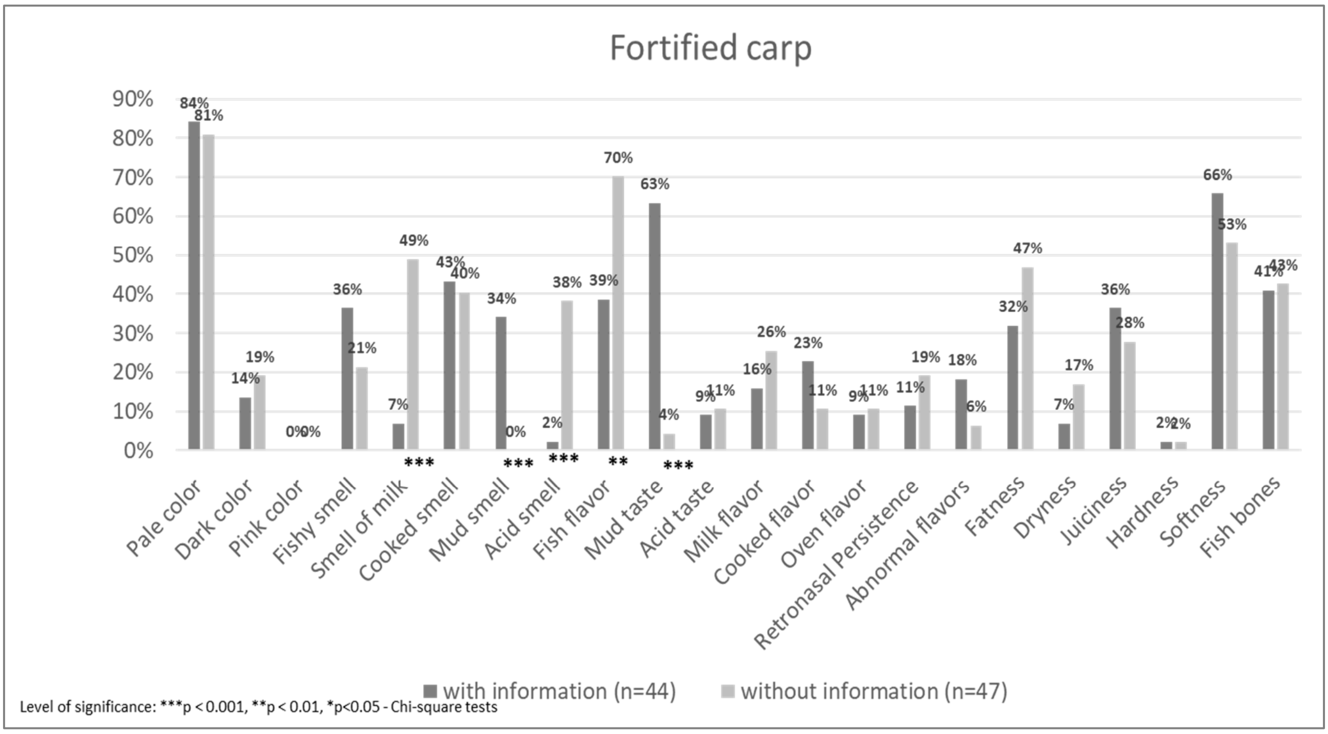

Supplementary Material File S3. Consumer evaluation of appearance, aroma, and taste of fortified and conventional fish with the absence or presence of information

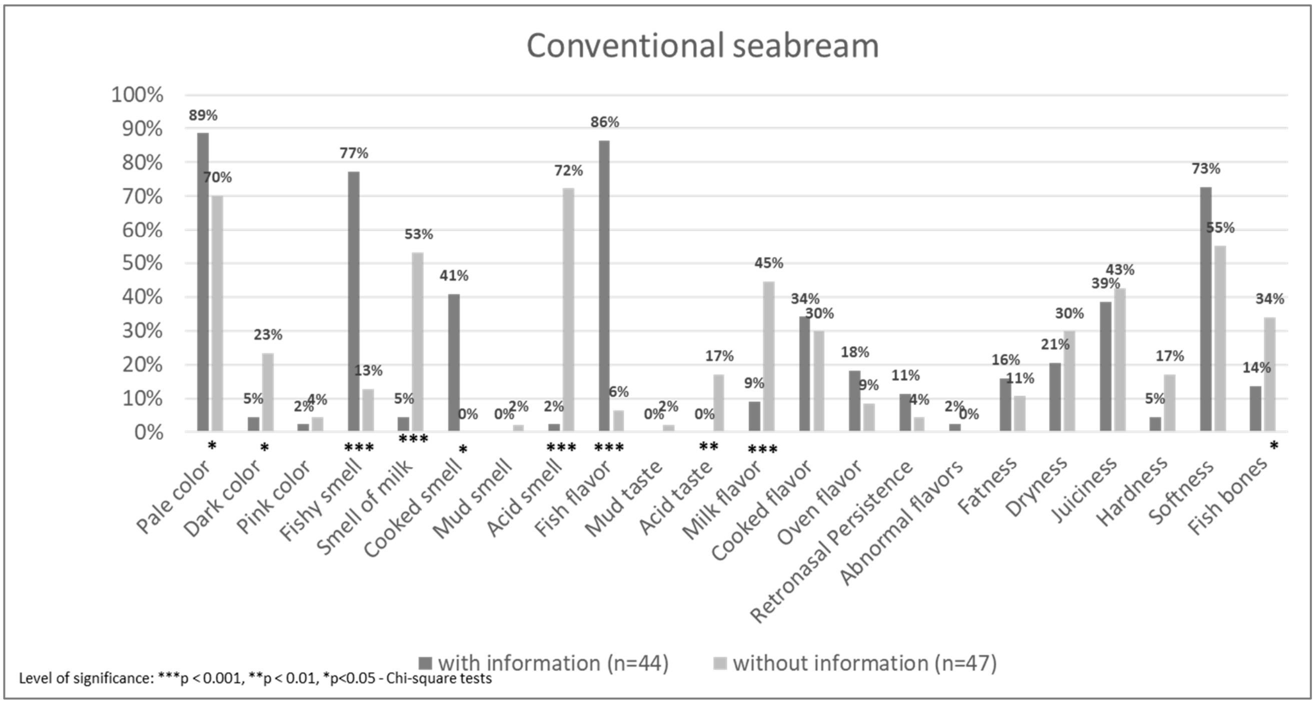

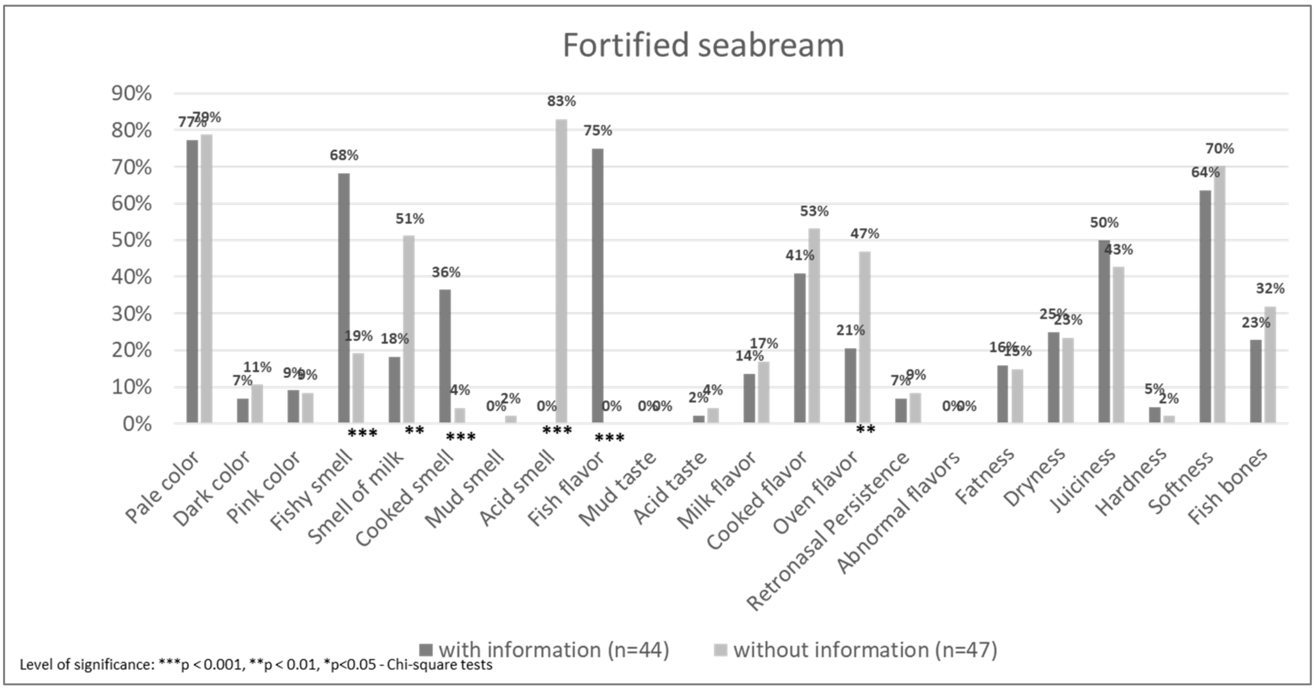

Supplement: Supplementary file 1 [file foods-11-02808-s001.zip › File S3.pdf]
